# VGLL3 is a prognostic biomarker and correlated with clinical pathologic features and immune infiltrates in stomach adenocarcinoma

**DOI:** 10.1038/s41598-020-58493-7

**Published:** 2020-01-28

**Authors:** Lihua Zhang, Longhai Li, Yong Mao, Dong Hua

**Affiliations:** 1 Department of Oncology, Affiliated Hospital of Jiangnan University, Jiangnan University, Wuxi, Jiangsu 214062 China; 2 School of Pharmaceutical Sciences, Jiangnan University, Wuxi, Jiangsu 214122 China; 3 Wuxi Medical College, Jiangnan University, Wuxi, Jiangsu 214122 China

**Keywords:** Prognostic markers, Gastric cancer, Outcomes research, Cancer genomics

## Abstract

Due to its poor clinical outcome, there is an urgent need to identify novel prognostic markers for stomach adenocarcinoma (STAD). Here, we aimed to explore the relationship between VGLL3 expression and clinico-pathological features, dendritic cells, macrophages, and prognosis of STAD. VGLL3 expression levels were significantly associated with histological grade, T stage, and TNM stage. VGLL3 levels and patient’s age were also independent prognostic factors of the clinical outcome of STAD. In addition, VGLL3 was associated with the abundance of macrophages and dendritic cells in tumor infiltrates, of which only VGLL3 and macrophage counts were the independent prognostic factors of immune cell infiltration in the TIMER Database. Extracellular matrix receptor interaction, focal adhesion, pathways in cancer, MAPK, JAK STAT, and WNT signaling pathways were enriched in VGLL3 high-expressing datasets as determined by Gene Set Enrichment Analysis (GSEA), while DNA replication, glyoxylate, and dicarboxylate metabolism, glutathione metabolism, homologous recombination, and glycosylphosphatidylinositol gpi banchor biosynthesis were enriched in VGLL3 low-expressing datasets. Thus, VGLL3 is a novel prognostic biomarker of both the clinical outcome and immune infiltration in STAD, and may therefore be a promising therapeutic target.

## Introduction

Despite advances in treatment modalities, gastric cancer remains the most common cause of cancer-related deaths in Asia, and the second most common cause of death worldwide^1^. Around 90% to 95% of gastric cancer cases involve stomach adenocarcinomas (STAD)^2^. Although the survival of STAD patients has greatly improved in the past 20 years due to surgery, chemotherapy and targeted therapy, the prognosis is still unsatisfactory^3^. Poor differentiation, late diagnosis, lack of predictive markers, and ineffective therapeutic targets are key factors that drive STAD metastasis and recurrence^4^. Therefore, it is essential to explore the molecular mechanisms underlying STAD development and progression to identify novel prognostic biomarkers and potential therapeutic targets to treat gastric cancer.

Transcription cofactor vestigial-like protein 3 (VGLL3) is a coactivator of mammalian toxicity equivalency factors (TEFs), and was associated with breast cancer, colon cancer, and lung cancer among other malignancies^5–9^. In our previous study, we found that VGLL3 at the protein level as determined by immunohistochemistry (IHC) was a novel prognostic biomarker for gastric cancer in the Chinese population^10^. However, its role at the mRNA level of STAD and immune microenvironment remains to be elucidated. To determine whether VGLL3 was involved in the progression of STAD, the Wilcox test and Kruskal test method were used to analyze the differences in VGLL3 expression in subgroups of clinic-pathological trait. Cox regression and log-rank test were used to evaluate significance of VGLL3 on disease prognosis.

Recently, more and more evidence showed immune cell infiltration plays a vital role in predicting overall survival in cancer such as lung cancer^11,12^, breast cancer^13^, bladder cancer^14^, pancreatic cancer^15^ and colon cancer^16^. Tanaka *et al*. emphasized the significance of immune-cell infiltration in gastric cancer^17^. In the immune cell infiltration, tumor-associated macrophages (TAM) was widely studied in cancers including gastric cancer^18–20^. Therefore, we hypothesized that VGLL3 expression was associated with immune cell infiltration and tumor-associated macrophages.

To determine any potential correlation of VGLL3 expression with immune cell infiltration of STAD, the Spearman’s correlation test was used to evaluate this relationship using TIMER databases. In addition, Cox regression, Kaplan-Meier survival analysis, and the log-rank test were used to determine the significance of VGLL3 expression on the prognosis of STAD with immune cell infiltration.

Furthermore, the potential underlying mechanism of VGLL3 in STAD was explored by using Gene Set Enrichment Analysis (GSEA). The gene sets were classified into VGLL3 high (VGLL3^hi^) and low expression (VGLL3^lo^) groups based on the median expression level. Kyoto Encyclopedia of Genes and Genomes (KEGG) pathways were significantly enriched by the criterium of false discovery rate (FDR) <0.05.

## Results

### Patient characteristics

Tumor samples were obtained from 317 patients who were pathologically diagnosed with STAD and were aged 62.16 ± 27.85 years; time to follow up of 10.19 years; 120 females and 197 males; grade of 7 Grade 1, 108 Grade 2 and 202 Grade 3; TNM stage 42 Stage I, 101 Stage II, 139 Stage III, and 35 Stage IV; T stage of 15 patients with tumors starting to grow into the wall of the stomach, 63 into the muscle layer, 152 into the outer lining of the stomach and 87 through the outer lining of the stomach; N stage of 99 patients with no lymph nodes containing cancer cells, 83 in 1 to 2 lymph nodes, 69 in 3 to 6 nearby lymph nodes, and 66 patients in 7 or more nearby lymph nodes; M stage of 295 patients with no metastasis and 22 metastasis. The patient’s general information is summarized in Table 1.

**Table 1:** Clinical and pathological characteristics of 317 gastric cancer patients.

Characteristics	Number of cases	
**Age**		
[0,44]	11	3.47%
[45,59]	90	28.39%
[60,74]	151	47.63%
[75,90]	65	20.50%
**Gender**		
Female	120	37.85%
Male	197	62.14%
**Grade**		
G1	7	2.21%
G2	108	34.07%
G3	202	63.72%
**TNM stage**		
Stage I	42	13.24%
Stage II	101	31.86%
Stage III	139	43.84%
Stage IV	35	11.04%
**T stage**		
T1	15	4.73%
T2	63	19.87%
T3	152	47.95%
T4	87	27.45%
**N stage**		
N0	99	31.23%
N1	83	26.18%
N2	69	21.76%
N3	66	20.82%
**M stage**		
M0	295	93.05%
M1	22	6.94%

### VGLL3 was associated with poor differentiation, advanced T stage, and TNM stage

**Figure 1:**
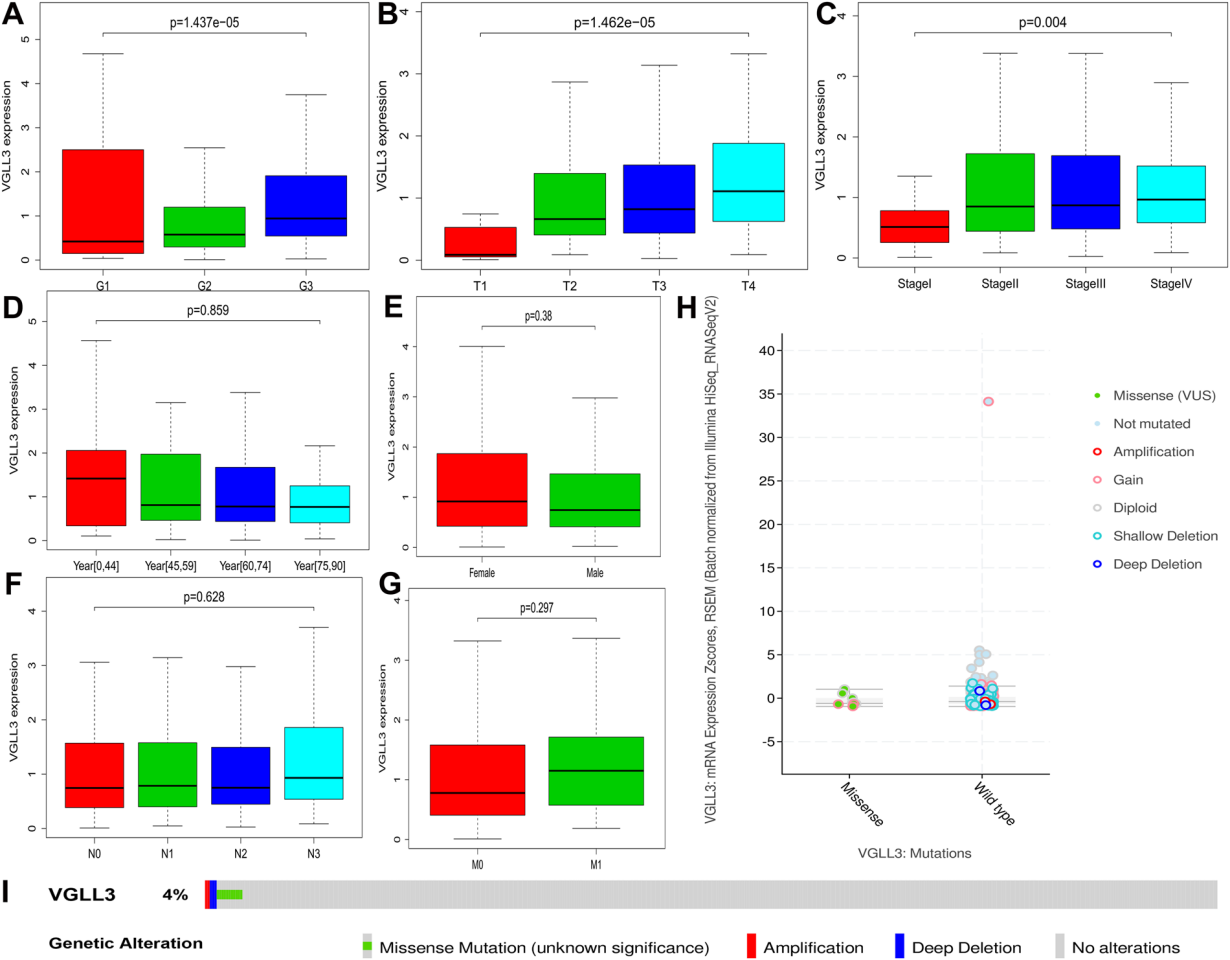
Correlation between VGLL3 expression and clinico-pathological features. Clinico-pathological features of STAD included (**A**) tumor differentiation grade (G1 - well differentiated, G2 - moderately differentiated, and G3 - poorly differentiated), (**B**) T stage, (**C**) TNM stage, (**D**) age, (**E**) gender, (**F**) regional lymph node metastasis, (**G**) distant metastasis, and (**H**) mutation status at transcription expression. Genetic alteration of VGLL3 at the DNA level (**I**).

To explore the correlationof VGLL3 on the progression of STAD, the Wilcox test and Kruskal test method were employed to analyze the difference in VGLL3 expression with clinico-pathological features. Our data showed that VGLL3 expression significantly correlated with the histological grade (P = 1.437e-05, Fig. 1A), T stage (P = 1.462e-05, Fig. 1B), and TNM stage (P = 0.004, Fig. 1C), but not with age, gender, N stage, and M stage (P > 0.05, Fig. 1D–G) of STAD. Taken together, these results showed that VGLL3 was highly expressed in poor differentiation (Fig. 1A), advanced T stage (Fig. 1B), and advanced TNM stage (Fig. 1C). Thus, these findings suggested that VGLL3 was involved in the progression of STAD. Considering that a mutation might result in loss of function, we mined the STAD mutation based on cBioPortal (www.cbioportal.org). There were only 8 missense mutation and 1 del mutation, which might result in loss of function in the 478 STAD samples (Fig. 1I). From the mutation status, no significant differences were observed in VGLL3 transcription expression between missense status and wild type status of VGLL3 (Fig. 1H). Thus, these data suggested that VGLL3 mutations were not associated with its expression.

### VGLL3 was an independent unfavorable prognostic marker with clinical features

**Figure 2:**
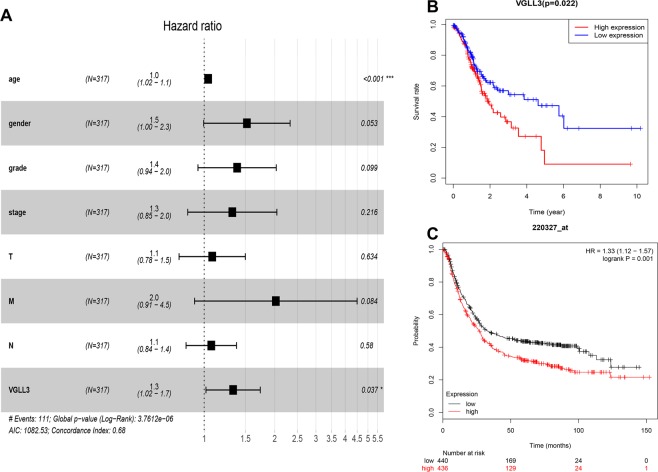
Correlation between VGLL3 expression and STAD prognosis in clinico-pathological features. (**A**) Multivariate Cox analysis showing the hazard ratios (HR) of different factors. The number of events for the number of tested factors was 111. The global p-value (Log-Rank) was 3.7612e-06; Akaike’s Information Criterion (AIC) was 1082.53; and the concordance index was 0.68. (**B**) Overall survival of VGLL3^hi^ and VGLL3^lo^ patients in the TCGA cohort. C. Overall survival of 440 VGLL3^hi^ and 436 VGLL3^lo^ patients in the Kaplan-Meier Plotter Database from GSE62254 (283), GSE14210 (119), GSE15459 (197), GSE22377 (43), GSE29272 (141), and GSE51105 (93) with 169 and 129 events respectively.

To explore the correlation of VGLL3 expression with the prognosis of STAD, the Cox proportional hazard regression model was employed to analyze prognostic factors. For the overall survival analysis, we used Kaplan-Meier survival analysis and log-rank test methods. Univariate Cox regression analysis showed that age, TNM stage, M stage, N stage, and VGLL3 levels were significantly associated with STAD prognosis (*P* < 0.05), however, gender and grade were not (Table 2). Multivariate Cox regression analysis indicated that only VGLL3 and age were independent prognostic factors (P < 0.01, Fig. 2A). In addition, Kaplan-Meier survival analysis showed that VGLL3 in the high expression group had a significantly poorer prognosis in the TCGA cohort (P < 0.05, Fig. 2B), which was validated in other cohorts in GEO (Gene Expression Omnibus) usingKaplan-Meier Plotter online data analysis P < 0.05, Fig. 2C).

**Table 2:** Univariate Cox proportional hazards analyses of overall survival of STAD patients.

Characteristics	HR	HR 95 L	HR 95 H	P value
Age	1.03	1.01	1.05	**0**.**006**
Gender	1.48	0.98	2.25	0.062
Grade	1.37	0.95	1.98	0.095
TNM stage	1.54	1.22	1.93	**0**.**000**
T stage	1.30	1.02	1.65	**0**.**032**
M stage	2.05	1.10	3.83	**0**.**025**
N stage	1.27	1.07	1.50	**0**.**006**
VGLL3 levels	1.33	1.05	1.69	**0**.**017**

Taken together, these results demonstrated that VGLL3 was an unfavorable prognostic factor and independent prognostic marker.

### VGLL3 associated with macrophages and dendritic cells

**Figure 3:**
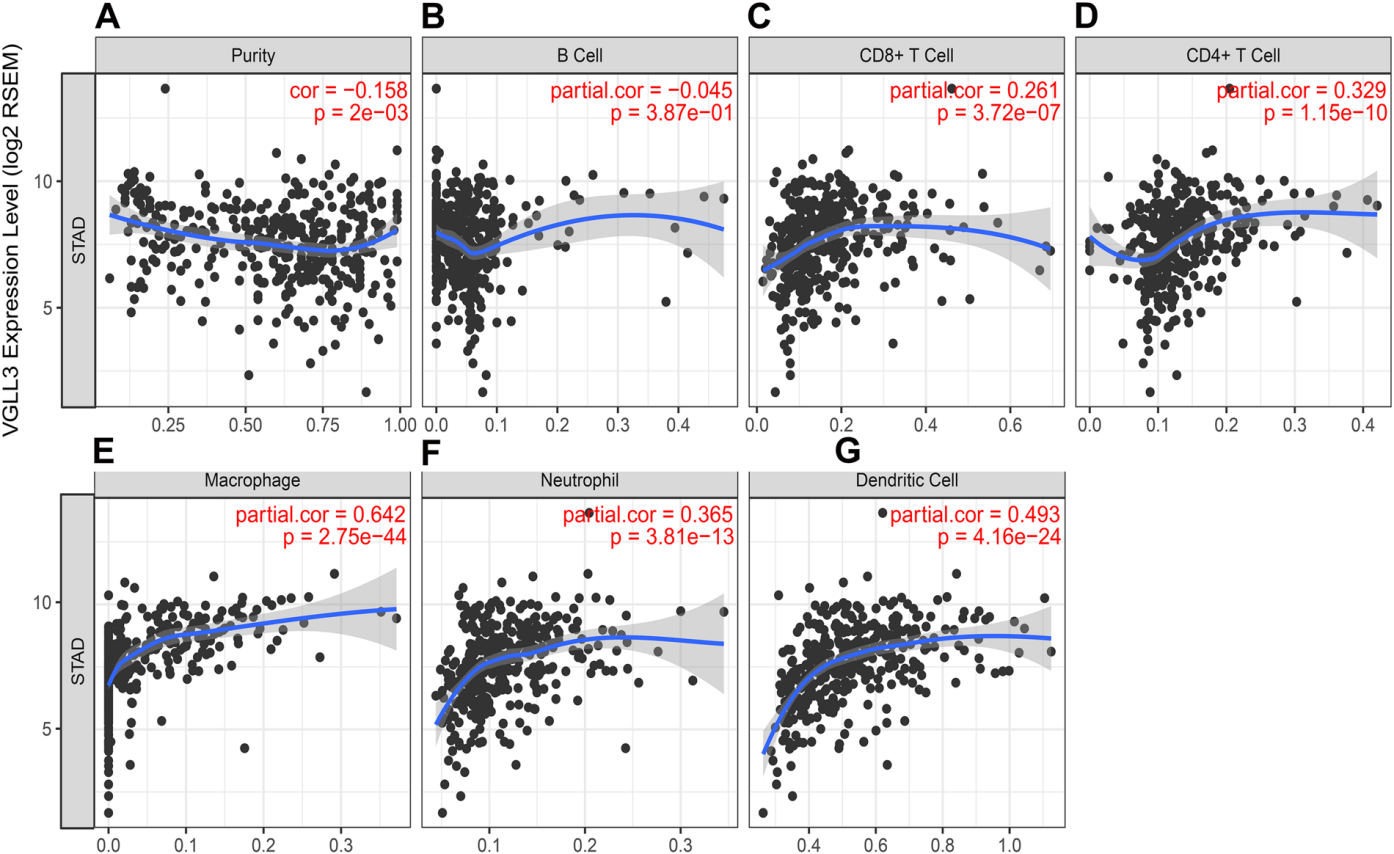
Correlation between VGLL3 expression and immune cell infiltration in STAD in the TCGA cohort. Tumor purity, (**B**) B cell abundance, (**C**) CD8^+^ T cells, (**D**) CD4^+^ T cells, (**E**) macrophages, (**F**) neutrophils, and (**G**) dendritic cells relative to VGLL3 expression.

STAD is a highly heterogeneous type of cancer, and factors such as the tumor microenvironment and immune cell infiltration can influence the prognosis^21^. Pearson correlation analysis showed a significant negative correlation between VGLL3 and tumor homogeneity (r = −0.158, *P* = 2e-03, Fig. 3A), and a positive correlation with CD8+ T cells (r = 0.261, *P* = 3.72e-07, Fig. 3C), CD4+ T cells (r = 0.329, *P* = 1.15e-10, Fig. 3D), macrophages (r = 0.642, *P* = 2.75e-44, Fig. 3E) neutrophils (r = 0.365, *P* = 3.81e-13, Fig. 3F) and dendritic cells (r = 0.493, *P* = 4.16e-24, Fig. 3G), but not with B cells (*P* > 0.05, Fig. 3B). Thus, these results suggested that VGLL3 maybe involved in macrophage and dendritic cell infiltrates.

### VGLL3 is an independent unfavorable prognostic marker with immune cell infiltration

**Figure 4:**
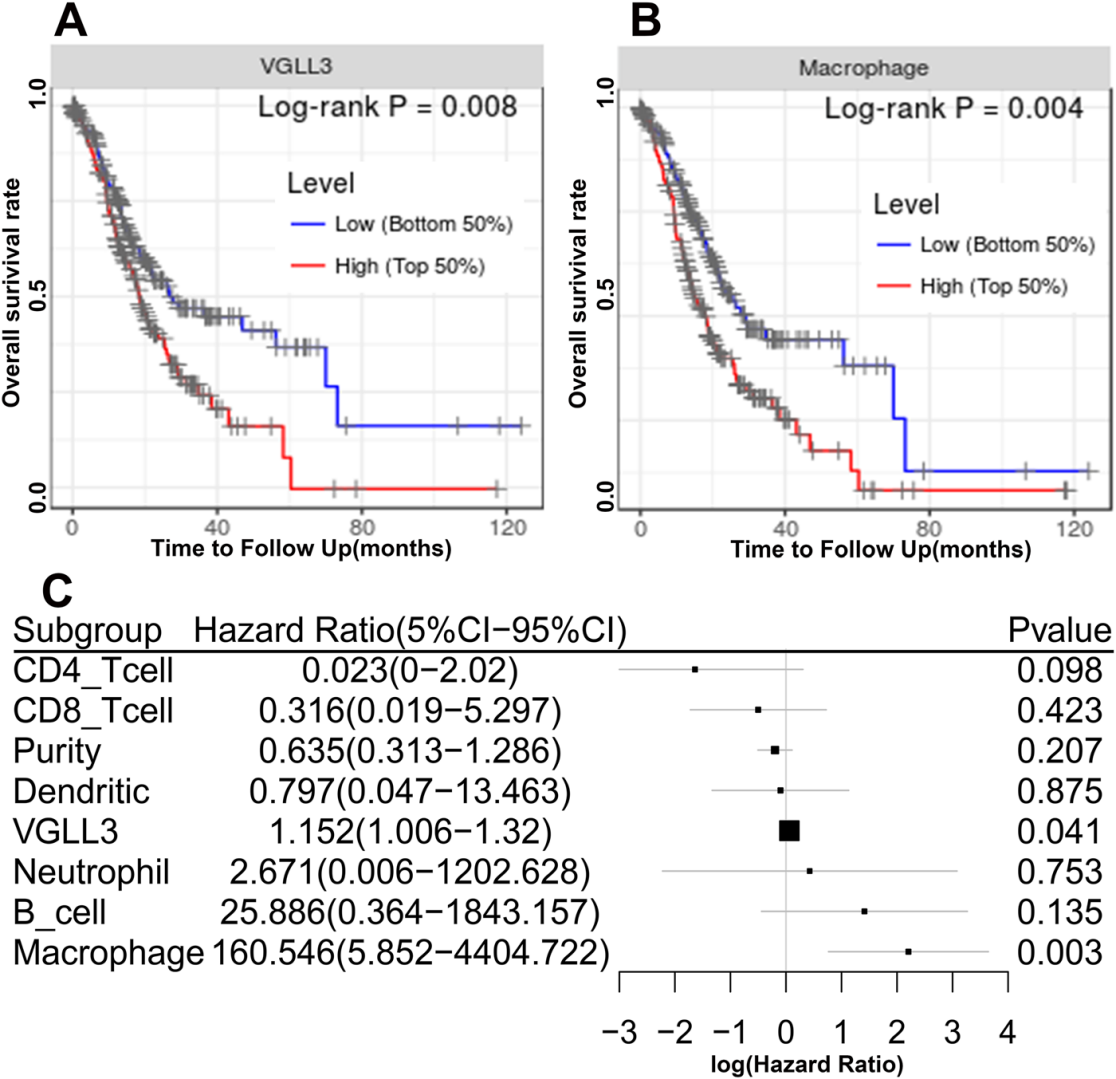
Correlation between VGLL3 expression and STAD prognosis in immune infiltration. (**A**,**B**) Kaplan-Meier survival analysis showing a significant association between macrophage infiltration. (**A**) VGLL3 levels and (**B**) overall survival. (**C**) Multivariate Cox analysis showing that VGLL3 and macrophage infiltration are independent prognostic factors of STAD.

To explore immune infiltrates and VGLL3 expression on the prognosis of STAD, Kaplan-Meier survival analysis and the log-rank test method were employed to analyze the overall survival. Both immune infiltrates and VGLL3 expression were divided into VGLL3 high and low levels by using the median expression. The Log-rank test and Kaplan-Meier survival analysis showed that high levels of VGLL3 (Fig. 4A) and macrophages (Fig. 4B) were significantly associated with poor survival (*P* < 0.05), whereas tumor purity, B cells, CD8^+^ T cells, CD4^+^ T cells, neutrophils, and dendritic cells were not (*P* > 0.05, data not shown).

Multivariate analysis further confirmed that VGLL3 and macrophages were independent prognostic factors (*P* < 0.05, Fig. 4C). Furthermore, VGLL3, neutrophils, B cells, and macrophage were unfavorable prognostic factors (Hazard ratio > 1, log (Hazard ratio) > 0), whereas others were found to be favorable prognostic factors (Hazard ratio < 1, log (Hazard ratio < 0). Thus, these data showed that VGLL3 was an independent unfavorable prognostic marker in immune infiltration.

### VGLL3 is associated with the PI3K/Akt/m TOR signaling pathway, extracellular matrix receptor interaction and focal adhesion

**Figure 5:**
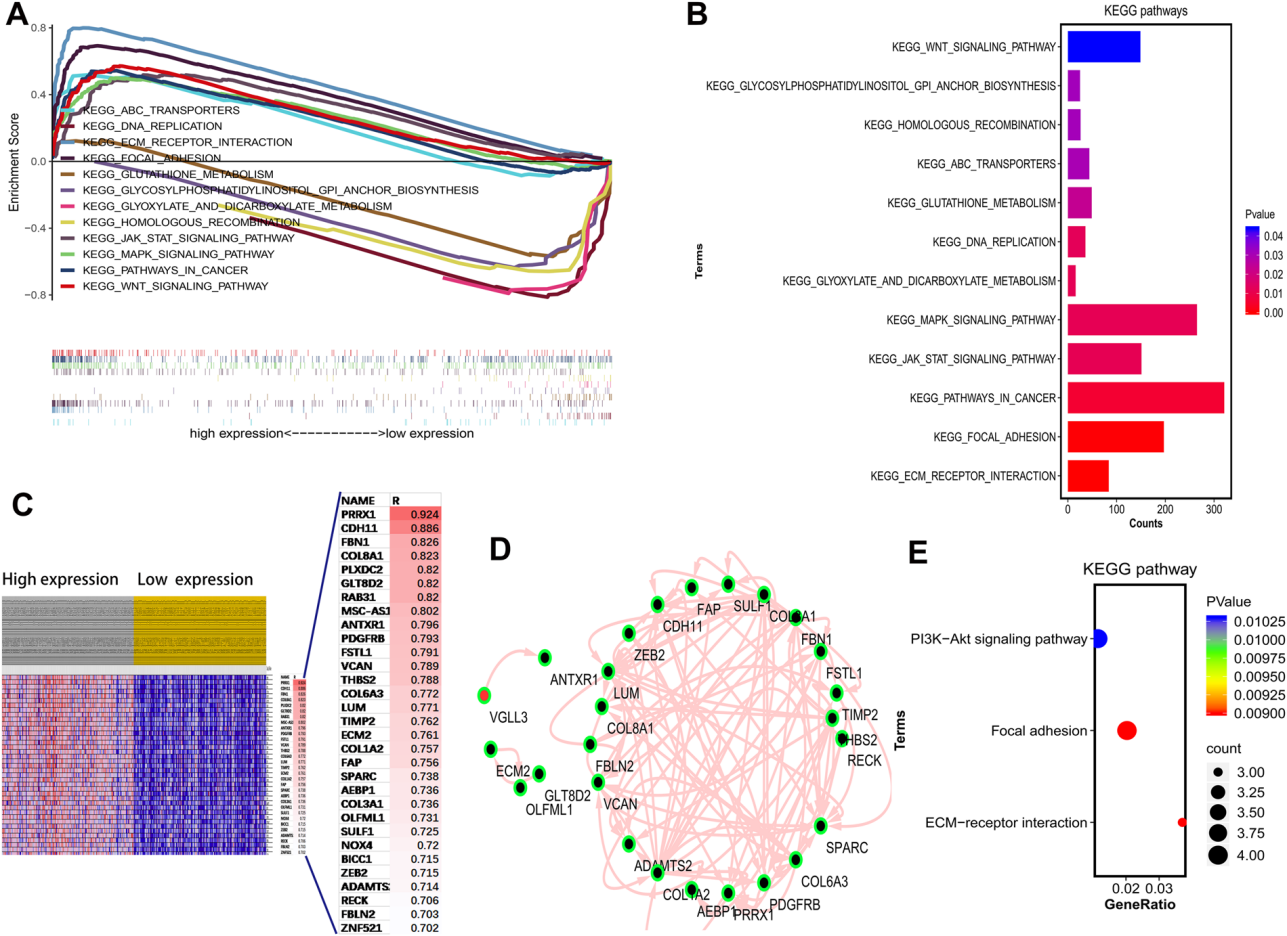
Gene set enrichment analysis of STAD datasets. (**A**) Differentially enriched pathways in VGLL3^hi^ and VGLL3^lo^ samples. (**B**) Significantly enriched pathways according to gene numbers and false discovery rate (FDR). (**C**) Heatmap of the top 31 VGLL3-associated genes. (**D**) Protein-protein-interaction (PPI) network of VGLL3 and co-expressing genes. (**E**) Enriched pathways of VGLL3-correlated genes.

To explore the potential underlying mechanism of VGLL3 in the progression of STAD, VGLL3 expression was first divided into high (VGLL3^hi^) and low (VGLL3^lo^) expression groups as determined by the median expression. Next, the gene set enrichment analysis (GSEA) method was employed to enrich Kyoto Encyclopedia of Genes and Genomes (KEGG) pathways. The gene sets associated with high VGLL3 expression were enriched in extracellular matrix (ECM) receptor interaction, focal adhesion, pathways in cancer, MAPK signaling pathway, JAK STAT signaling pathway, ABC transporters, and the WNT signaling pathway. In contrast, VGLL3 low expression-related gene sets were enriched in glyoxylate and dicarboxylate metabolism, DNA replication, glutathione metabolism, homologous recombination and glycosylphosphatidylinositol gpi banchor biosynthesis (Fig. 5A,B). To further investigate the molecular mechanism underlying VGLL3 expression, a heat map of the top 31 genes was created, which showed a strong correlation with VGLL3 expression based on r > 0.7 (Fig. 5C). Protein-protein interaction (PPI) network analysis between VGLL3 expression and co-expressed genes was determined by building a regulatory network using Cytoscape software, and was analyzed using STRING (https://string-db.org/). The crosstalk between genes (Fig. 5D) further showed a significant enrichment of the PI3K/Akt/m TOR signaling pathway, ECM receptor interaction and focal adhesion (Fig. 5E). Together, these results demonstrated that VGLL3 was involved in cancer-related KEGG pathways.

It might be that VGLL3 plays a vital role in the progression of STAD by regulating the PI3K/Akt/m TOR signaling pathway, ECM receptor interaction, and focal adhesion.

## Discussion

VGLL3 is an inhibitor of adipocyte differentiation and a novel Ets1 interacting partner, and regulates trigeminal nerve formation and cranial neural crest migration^7,22^. In cancer, VGLL3 has been shown to be overexpressed in a subset of soft tissue sarcomas^8^, and was associated with a tumor suppressor phenotype in epithelial ovarian cancer^6^. When studying the association of VGLL3 and the progression of STAD, we analyzed VGLL3 expression levels in STAD samples from the TCGA dataset, and found that high levels of VGLL3 positively correlated with a higher histological grade, T stage, and TNM stage of the tumor, which are indicators of greater tumor malignancy^23^. In addition, VGLL3 overexpression was also associated with worse prognosis and poor overall survival. Moreover, multivariate Cox regression analysis confirmed VGLL3 as an independent unfavorable prognostic factor of STAD. Together, these results demonstrated that VGLL3 might promote theprogression of STAD^6^. In contrast, VGLL3 acts as a tumor suppressor in epithelial ovarian cancer. Thus, an effective marker to predict prognosis in cancer patients is urgently needed to optimize tailored treatment in precision medicine. This will aid in individualized treatments, postoperative counseling, and result in improved survival.

STAD is a highly heterogeneous cancer, and tumor heterogeneity is instrumental in immune evasion^24–28^. In fact, several studies have shown that tumor infiltrating immune cells are prognostic markers for cancer progression^29–31^. Therefore, we hypothesized that VGLL3 expression is associated with immune infiltration in STAD tumors, and a potential marker of the tumor immune microenvironment. CIBERSORT is an analytical tool developed to estimate the abundance of member cell types in a mixed cell population^32^. Pearson correlation analysis has been performed between genes and immune cell infiltration determined by both CIBERSORT^33^ and TIMER. However, for this analysis we used TIMER because of its easy to use web interfaceand its abundant incorporatation of clinical data. In our study, we found that VGLL3 expression positively correlated with tumor heterogeneity regarding immune cell infiltration, particularly with CD8+ T cells, CD4+ T cells, macrophages, neutrophils, and dendritic cells, but not B cells. This suggested that VGLL3 may activate or inhibit immune cells infiltrating the tumor, although the underlying mechanism involved is unknown. Univariate analysis showed that T cells and dendritic cells were favorable prognostic factors, while VGLL3 levels, neutrophils, B cells, macrophages, and tumor heterogeneity were detrimental prognostic factors. Therefore, it is possible that VGLL3 inhibits T cells and dendritic cells, and activates neutrophils and macrophages. Multivariate analysis showed that along with VGLL3 levels, macrophages were also an independent prognostic factor. Tumor-associated macrophages (TAM) affect several aspects of tumor physiology, including tumor cell proliferation^34^, angiogenesis^35^, invasion^36^, metastasis^37^, immunosuppression^38^, and drug resistance^39^. In a previous study, TAM were reported as a biomarker for gastric cancer^18,40^, and TAM were strongly associated with VGLL3 expression in the current study. This suggested that immunotherapy might be considered based on VGLL3 expression.

To further elucidate the molecular mechanisms underlying the role of VGLL3 in STAD, we searched for pathways enriched in VGLL3 overexpressing datasets using GSEA. We found that high VGLL3 levels significantly associated with ECM receptor interaction, focal adhesion, pathways in cancer, MAPK signaling pathway, JAK STAT signaling pathway, ABC transporters, and the WNT signaling pathway, and that low expression levels associated with glyoxylate and dicarboxylate metabolism, DNA replication, glutathione metabolism, homologous recombination, and glycosylphosphatidylinositol gpi banchor biosynthesis. Given that VGLL3 is a coactivator of mammalian TEFs, it likely regulates more genes and pathways indirectly. Although there were several gene sets in the GSEA, here we focused on the KEGG pathway gene set to elucidate the molecular mechanism of VGLL3, which might affect one or several KEGG signaling pathways. Therefore, we next analyzed the pathways enriched in genes that strongly correlated with VGLL3 expression (r > 0.7), and identified the PI3K/Akt/mTOR signaling pathway, ECM receptor interaction, and focal adhesion pathway. The PI3K/AKT/mTOR signaling pathway plays a significant role in tumor cell proliferation, growth and survival, in addition to modulating the tumor microenvironment and tumor-associated macrophages (TAMs)^41–47^. ECM and focal adhesion molecules, including PLXDC2, PDGFRB, FSTL1, TIMP2, FAP, SPARC, AEBP1, NOX4, and FBLN2 are known to regulate macrophage mobilization into tumor tissues^19,20,48–50^, and are associated with TAM^51–53^. Therefore, it is possible that VGLL3 also modulates TAM via at least some of those genes.

Considering that mutations might result in loss of function, we mined the STAD mutations using the cBioPortal (www.cbioportal.org). There were 8 missense mutations and 1 del mutation, which might result in loss of function. The VGLL3 associated mutation rate was 1.8% in 478 samples with STAD. Due to the low mutation rate, the effect of the VGLL3 mutation status could be neglected.

To summarize, VGLL3 has a potential prognostic and therapeutic value in STAD, because its overexpression was associated with advanced tumor stage, poor differentiation, TAM infiltration, and poor prognosis. The enriched pathways and co-expressing genes identified in this study provide further insights into the molecular basis of the role of VGLL3 in STAD. Overall, our findings show that VGLL3 plays a significant role in the prognosis and clinical progression of STAD, as well as in immune cell infiltration. However, since the TCGA database contains mRNA expression data and not *in situ* protein expression data, our findings will have to be validated in patient’s tissue samples using immunohistochemistry, and correlated to their clinico-pathological features.

## Materials and Methods

### RNA-sequencing data and samples

The RNA-sequencing V2 datasets and clinical data of STAD samples were downloaded using the Bioconductor/TCGA Biolinks function package from the TCGA database (http://tcga-data.nci.nih.gov./tcga/)^54^.

### Correlation analysis of VGLL3 and clinico-pathological features

The differences in VGLL3 expression at different stages of clinico-pathological features were evaluated by the Wilcox test and Kruskal test. P-values < 0.05 were considered statistically significant.

### Significantly prognostic marker analysis

R.3.5.1 version software was used for all statistical analyses, with limma, beeswarm, survival, survminer, and ggplot2 packages as appropriate. Univariate Cox regression analyses and multivariate Cox analysis were employed to identify independent prognostic factors. *P*-values < 0.05 were considered statistically significant.

### Overall survival analysis

Kaplan-Meier survival analysis was used to create survival curves, which were compared by log-rank tests. The correlation between VGLL3 expression and survival was analyzed using the Kaplan-Meier plotter (http://kmplot.com/analysis/), which was based on the HGU133 Plus 2.0 array data of 1,065 STAD patients with a mean follow-up of 33 months. In addition, the hazard ratio (HR) with 95% confidence intervals and log-rank P-value were computed.

### Correlation analysis of VGLL3 and immune cell infiltration

The abundance of tumor-infiltrating immune cells (TIICs) in STAD was predicted using the TIMER web tool (https://cistrome.shinyapps.io/timer/) using data from TCGA to estimate the abundance of immune infiltrates^55^. The correlation between VGLL3 expression and the abundance of different immune cells, including CD4+ T cells, CD8+ T cells, B cells, neutrophils, macrophages, and dendritic cells was analyzed using the Spearman’s correlation test.

### Functional KEGG pathway enrichment analysis

The STAD-associated gene clusters and pathways were identified in the c2.cp.kegg.v6.2.symbols symbols.gmt data set from the Msig database using Gene set enrichment analysis (GSEA) version 3.0. Furthermore, enrichment analysis was performed with random combination number of 1000 and false discovery rate (FDR) < 0.05 as the criteria for significantly enriched genes. Gene sets were classified into VGLL3 high- and low-expression groups as based on the median VGLL3 expression level, and the effect of VGLL3 expression was evaluated. Finally, the crosstalk between VGLL3 and co-expressed genes was determined by building a regulatory network using Cytoscape software, and analyzed using STRING (https://string-db.org/). In the Spearman’s test, correlation coefficients ranging from 0.7–1 indicated a “very strong” correlation. *P*-values < 0.05 were considered statistically significant.
